# Association of non-high-density lipoprotein cholesterol to high-density lipoprotein cholesterol ratio with coronary heart disease: Establishment and validation of a clinical nomogram model

**DOI:** 10.1097/MD.0000000000041896

**Published:** 2025-03-14

**Authors:** Wenlong Ding, Tao Li, Caoyang Fang, Xin-Xin Zhang, Enyang Wang

**Affiliations:** a Department of Cardiology, Xuancheng Hospital Affiliated to Wannan Medical College (Xuancheng People’s Hospital), Xuancheng, Anhui, China; b Department of Geriatrics, Mengcheng First People’s Hospital, Mengcheng, Anhui, China; c Department of Emergency, First Affiliated Hospital of University of Science and Technology of China, Anhui Provincial Hospital, Hefei, Anhui, China; d Department of Cardiology, The Second People’s Hospital of Hefei, Hefei Hospital Affiliated to Anhui Medical University, Hefei, Anhui, China.

**Keywords:** CHD, coronary heart disease, NHHR, nomogram, predicted

## Abstract

The purpose of this study is to investigate the non-high-density lipoprotein cholesterol (non-HDL-C) to high-density lipoprotein cholesterol (HDL-C) ratio (NHHR) as a novel compound lipid index for atherosclerosis and explore its relationship with coronary heart disease (CHD). This study also aims to establish NHHR as a sensitive indicator for early prevention of CHD and to construct a clinical prediction model to further predict the occurrence of CHD. This study selected 707 patients who visited the First People’s Hospital of Mengcheng County from January 2020 to May 2024, including 466 patients with CHD and a control group. Logistic regression analysis was used to analyze the correlation between NHHR and CHD. Patients were randomly divided into a training set and validation set in a 7:3 ratio. Multivariable logistic regression was used to screen for risk factors, and a nomogram model was constructed and validated. After adjusting for confounding factors, the results showed that for each increase of 1 standard deviation in NHHR, the risk of CHD increased by 42%, with a *P*-value of .003. In model 3, the risk of CHD for the highest quartile increased by 144%, with a *P*-value of .01. The smoothed curve fitting showed a nonlinear relationship between NHHR and CHD. Multivariable logistic analysis indicated that age, body mass index, smoke, hypertension, white blood cells, fasting plasma glucose, uric acid, and NHHR were independent risk factors for predicting the occurrence of CHD (*P* < .05), and a risk prediction nomogram model was constructed. The receiver operating characteristic curve analysis of the training set showed an AUC of 0.922 (95% CI: 0.900–0.945), and the AUC of the validation set was 0.902 (95% CI: 0.856–0.948), indicating good model accuracy. Calibration curve analysis showed that the calibration curves of the nomogram model were very close for predicting the occurrence of CHD in the training set and validation set, and the decision curve analysis also showed a good clinical net benefit of the nomogram model. The study results indicated a strong and nonlinear correlation between NHHR and CHD. Our constructed nomogram model has a certain predictive ability for the occurrence of CHD.

## 1. Introduction

Previous research indicates that low-density lipoprotein cholesterol (LDL-C) is significantly associated with atherosclerosis, making it the primary focus of lipid-lowering treatments. However, even with LDL-C levels managed, the rates of atherosclerotic cardiovascular disease (ASCVD) remain elevated. Consequently, non-high-density lipoprotein cholesterol (non-HDL-C) has gained attention as a valuable indicator. Studies have demonstrated that non-HDL-C serves as a more effective predictor and prevention target for cardiovascular events and atherosclerosis than LDL-C.^[1,2]^ In 2016, the ACC Expert Consensus Committee suggested that non-HDL-C could be considered an equivalent measure to LDL-C for patients with diabetes and high triglyceride levels.^[3]^ The following year, the ACC Expert Consensus Committee Working Group revised its guidelines regarding the use of non-statin medications in preventing coronary artery disease,^[4]^ positioning non-HDL-C as a target for all risk categories. Recently, the non-HDL-C/HDL-C ratio has garnered increased interest, as it is thought to represent mixed dyslipidemia and offers a superior assessment of the balance between atherosclerosis and anti-atherosclerosis compared to traditional lipid profiles. Numerous studies have linked the non-HDL-C/HDL-C ratio to various lipid-related conditions, including atherosclerotic plaques,^[5]^ metabolic syndrome,^[6]^ insulin resistance,^[7]^ nonalcoholic fatty liver disease,^[8]^ chronic kidney disease,^[9]^ and coronary heart disease (CHD).^[10]^ Notably, some research suggests that the non-HDL-C/HDL-C ratio is a stronger independent predictor of CHD progression, the severity of CAD, and MACE compared to individual lipid parameters like LDL-C, HDL-C, and non-HDL-C.

The purpose of this study is to investigate the correlation between non-high-density lipoprotein cholesterol/high-density lipoprotein cholesterol ratio (NHHR) and the occurrence of CHD in order to find a more sensitive indicator for early prevention. Additionally, a clinical prediction model will be established based on NHHR to provide more effective treatment reference for patients.

## 2. Materials and methods

### 2.1. Study subjects and groups

This study randomly selected 707 patients admitted to the First People’s Hospital of Mengcheng County from January 2020 to May 2024, including 531 males and 176 females, with a median age of 62.00 (52.00, 71.00) years. The inclusion criteria were: complete medical records, with all enrolled patients undergoing coronary angiography and blood lipid index testing. According to the internationally recognized diameter method, the coronary angiography showing at least 1 coronary artery vessel luminal diameter narrowed by ≥50% was defined as CHD, and those without significant abnormalities were considered non-CHD patients and serve as the control group; all patients signed a written informed consent form. Exclusion criteria were: presence of other serious cardiac diseases such as heart failure, cardiomyopathy, and severe arrhythmias; presence of other serious non-cardiovascular diseases such as advanced liver disease, renal failure, malignant tumors, and severe infection; severe cognitive impairment or intellectual disability; and patients with a maximal narrowing diameter of any vessel luminal diameter < 50% shown by coronary angiography. Based on the presence or absence of CHD, patients were divided into a CHD group (n = 466) and a non-CHD control group (n = 241). This study has been approved by the Ethics Committee of the First People’s Hospital of Mengcheng County.

### 2.2. Method

#### 2.1.1. Data collection and processing.

 This study used a uniform design of health information questionnaire and an electronic medical record system to collect patients’ clinical data, including demographic data (such as age, gender, BMI, smoking history, alcohol intake history, history of hypertension, and history of diabetes) and laboratory indicators (such as white blood cells (WBC), lymphocytes, red blood cells, platelets, fasting blood glucose (FPG), creatinine, uric acid (UA), blood urea nitrogen, triglycerides (TG), total cholesterol (TC), low-density lipoprotein cholesterol (LDL-C), and high-density lipoprotein cholesterol (HDL-C)). In collecting patients’ clinical data, we performed data preprocessing by deleting outliers and errors and used the “MICE” imputation method to fill in missing values to ensure the accuracy and completeness of the dataset.

#### 2.1.2. Definition of NHHR.

 NHHR is an index calculated based on the serum levels of TC and HDL-C in patients. Non-HDL-C refers to the value obtained by subtracting HDL-C from TC, and NHHR is the calculated ratio of Non-HDL-C to HDL-C.^[11]^ We divided patients into 4 groups (Q1, Q2, Q3, and Q4) based on the quartiles of NHHR, and Q1 group was selected as the reference group.

#### 2.1.3. Statistical analysis.

 Statistical analysis was performed using R software (Version 4.2.1) and SPSS 26.0 (Chicago). For normally distributed continuous variables, the mean ± standard deviation was used, and independent sample t-tests were used for comparisons between 2 groups. For non-normally distributed continuous variables, the median M (P25, P75) was used, and the Mann–Whitney U test was used for comparisons between groups. For categorical data, the rate was used, and the chi-square test was used for comparisons between 2 groups. Prior to the above statistical analyses, normality of continuous variables was assessed using the Kolmogorov–Smirnov test.

We used multivariable logistic regression analysis to identify independent predictors affecting the occurrence of CHD (refer to Table 1), and to investigate the relationship between NHHR and CHD. To achieve this, we divided patients into 4 groups (Q1, Q2, Q3, Q4) based on the quartiles of NHHR, with Q1 group serving as the reference group. We used a weighted logistic regression model to analyze the association between NHHR and CHD. In Model 1, we conducted no covariate adjustment, while in Model 2, we adjusted for age, BMI, smoke, and hypertension. In Model 3, we further adjusted for age, BMI, smoke, hypertension, WBC, UA, and FPG. Finally, we performed a trend analysis. In addition, we used a generalized additive model with a spline smoothing function to verify the linearity of the observed correlation in the quartile analysis, and used the likelihood ratio test to examine the significance of the linear relationship.

**Table 1:** Univariate and multivariate logistic regression analysis.

Variables	Univariate logistic regression analysis					Multivariate logistic regression analysis				
	β	SE	*Z*	*P*	OR (95% CI)	β	SE	*Z*	*P*	OR (95% CI)
Sex										
Female					1.00 (reference)					1.00 (reference)
Male	0.52	0.18	2.92	.003	1.69 (1.19–2.39)	−0.20	0.34	−0.61	.544	0.82 (0.42–1.58)
Smoke										
No					1.00 (reference)					1.00 (reference)
Yes	1.02	0.17	6.14	<.001	2.78 (2.00–3.84)	1.25	0.29	4.39	<.001	3.49 (2.00–6.11)
Diabetes										
No					1.00 (reference)					1.00 (reference)
Yes	1.14	0.24	4.84	<.001	3.13 (1.97–4.97)	0.46	0.35	1.30	.193	1.58 (0.79–3.14)
Alcohol										
No					1.00 (reference)					
Yes	0.15	0.19	0.80	.426	1.16 (0.80–1.70)					
Hypertension										
No					1.00 (reference)					1.00 (reference)
Yes	−0.48	0.17	−2.92	.003	0.62 (0.45–0.85)	−0.64	0.25	−2.54	.011	0.53 (0.32−0.86)
Age	0.01	0.01	2.07	.039	1.01 (1.01–1.03)	0.05	0.01	4.78	<.001	1.06 (1.03–1.08)
BMI	−0.07	0.02	−3.01	.003	0.93 (0.88–0.97)	0.10	0.04	2.61	.009	0.90 (0.84–0.97)
WBC	0.65	0.05	12.32	<.001	1.91 (1.73–2.12)	0.72	0.06	11.09	<.001	2.06 (1.81–2.33)
RBC	0.06	0.14	0.41	.684	1.06 (0.81–1.39)					
Platelets	−0.00	0.00	−0.42	.673	1.00 (1.00–1.00)					
FPG	0.36	0.05	6.48	<.001	1.43 (1.28–1.59)	0.11	0.05	2.23	.026	1.11 (1.01–1.23)
BUN	0.04	0.03	1.16	.245	1.04 (0.97–1.11)					
Creaintine	0.01	0.00	2.31	.021	1.01 (1.01–1.01)	0.01	0.01	1.12	.262	1.01 (0.99–1.02)
UA	−0.01	0.00	−2.60	.009	0.99 (0.99–0.99)	−0.01	0.00	−4.29	<.001	0.99 (0.99–0.99)
TG	0.03	0.04	0.65	.515	1.03 (0.95–1.11)					
LDL	0.04	0.09	0.39	.698	1.04 (0.87–1.24)					
NHHR	0.22	0.07	2.95	.003	1.24 (1.08–1.43)	0.34	0.12	2.97	.003	1.41 (1.12–1.77)
Lymphocytes	0.06	0.08	0.68	.494	1.06 (0.90–1.25)					

BMI = body mass index, BUN = blood urea nitrogen, FPG = fasting plasma glucose, LDL = low-density lipoprotein, NHHR = non-HDL cholesterol to HDL cholesterol ratio, RBC = red blood cell, TG = triglyceride, UA = uric acid, WBC = white blood cell.

Based on the predictors obtained from the above multivariable logistic regression analysis, we constructed a column chart model to predict the risk of CHD occurrence, and evaluated the predictive performance of the model using the receiver operating characteristic curve (ROC) curve. We also evaluated the calibration of the model by drawing a calibration curve. In addition, we plotted a clinical decision curve to analyze the net benefit rate of the column chart model for predicting the occurrence of CHD.

## 3. Results

### 3.1. Characteristics of study participants

Table 2 presents the basic characteristics of the participants. The average age of the study population was 62.00 (52.00, 71.00) years, including 531 males and 176 females. Compared with non-CHD participants, CHD participants showed statistically significant differences in BMI, WBC, lymphocytes, FPG, NHHR, gender, smoking history, diabetes history, hypertension history, and other factors (*P* < .05). However, no statistically significant differences were observed in other indicators (*P* > .05).

**Table 2:** General clinical characteristics of study participants.

Variables	Total (n = 707)	Non-CHD (n = 241)	CHD (n = 466)	*t/Z/*χ^2^	*P*
RBC, mean ± SD	4.46 ± 0.57	4.45 ± 0.47	4.47 ± 0.62	−0.44	.659
Age, M (Q1, Q3)	62.00 (52.00–71.00)	62.00 (52.00, 69.00)	62.00 (52.00, 72.75)	−1.64	.101
BMI, M (Q1, Q3)	24.70 (22.47–27.00)	24.75 (22.94, 27.36)	24.70 (22.00, 26.98)	−2.45	.014
WBC, M (Q1, Q3)	8.54 (6.38–11.37)	6.07 (4.90, 7.73)	10.14 (7.99, 12.66)	−16.14	<.001
Lymphocytes, M (Q1, Q3)	1.55 (1.10–2.09)	1.65 (1.31, 1.99)	1.44 (1.04, 2.16)	−2.31	.021
Platelets, M (Q1, Q3)	197.00 (156.00–239.50)	192.00 (155.00, 243.00)	197.00 (158.00, 239.00)	−0.53	.598
FPG, M (Q1, Q3)	5.79 (5.08–7.37)	5.14 (4.81, 5.54)	6.41 (5.36, 8.32)	−11.31	<.001
BUN, M (Q1, Q3)	5.45 (4.43–6.75)	5.45 (4.60, 6.60)	5.47 (4.35, 6.85)	−0.36	.716
Creaintine, M (Q1, Q3)	69.00 (58.75–83.90)	66.90 (58.30, 83.90)	70.55 (59.00, 83.00)	−1.40	.161
UA, M (Q1, Q3)	357.60 (294.90–427.55)	366.80 (302.00, 441.80)	353.50 (286.00, 421.60)	−1.82	.069
TG, M (Q1, Q3)	1.43 (1.01–2.07)	1.44 (1.01–1.97)	1.43 (1.02, 2.13)	−0.03	.973
LDL, M (Q1, Q3)	2.77 (2.24–3.39)	2.79 (2.35–3.33)	2.75 (2.23, 3.41)	−0.21	.836
NHHR, M (Q1, Q3)	3.07 (2.34–3.73)	2.92 (2.20–3.65)	3.13 (2.47, 3.82)	−2.97	.003
Sex, n (%)					
Female	176 (24.89)	76 (31.54)	100 (21.46)	8.63	0.003
Male	531 (75.11)	165 (68.46)	366 (78.54)		
Smoke, n (%)					
No	360 (50.92)	162 (67.22)	198 (42.49)	38.87	<.001
Yes	347 (49.08)	79 (32.78)	268 (57.51)		
Alcohol, n(%)					
No	545 (77.09)	190 (78.84)	355 (76.18)	0.64	0.425
Yes	162 (22.91)	51 (21.16)	111 (23.82)		
Diabetes, n (%)					
No	558 (78.93)	216 (89.63)	342 (73.39)	25.17	<.001
Yes	149 (21.07)	25 (10.37)	124 (26.61)		
Hypertension, n (%)					
No	288 (40.74)	80 (33.20)	208 (44.64)	8.61	.003
Yes	419 (59.26)	161 (66.80)	258 (55.36)		

BMI = body mass index, BUN = blood urea nitrogen, FPG = fasting plasma glucose, LDL = low-density lipoprotein, NHHR = non-HDL cholesterol to HDL cholesterol ratio, RBC = red blood cell, TG = triglyceride, UA = uric acid, WBC = white blood cell.

### 3.2. Comparison of baseline data and laboratory test indicators between training set and validation set

Table S1, Supplemental Digital Content (http://links.lww.com/MD/O536), lists the basic characteristics of the participants in the training and validation sets. There were statistically significant differences between the 2 groups of patients in WBC and FPG indicators (*P* < .05), while no statistically significant differences were observed in other indicator comparisons (*P* > .05).

### 3.3. Relationship between NHHR and CHD

In Table 3, we summarized the data on the correlation between NHHR and CHD. We found that for Model 1, with each unit increase in NHHR, the risk of CHD increased by 24% (OR: 1.24, 95% CI: 1.08–1.44, *P* = .003). For Model 2, with each unit increase in NHHR, the risk increased by 39% (OR: 1.39, 95% CI: 1.19–1.64, *P* < .0001). In Model 3, after further adjustment, with each unit increase in NHHR, the risk increased by 42% (OR: 1.42, 95% CI: 1.13–1.79, *P* = .003). In addition, in the quartile analysis, when comparing the highest quartile of NHHR with the lowest quartile of the entire model, it was found that the risk of CHD increased by 144% (OR: 2.44, 95% CI: 1.23–4.92, *P* = .01). Moreover, there was a trend of increasing CHD risk from Q1 to Q4 (*P* = .015).

**Table 3:** Correlation analysis between NHHR and CHD.

	Model 1		Model 1		Model 1	
	OR (95% CI)	*P*	OR (95% CI)	*P*	OR (95% CI)	*P*
NHHR	1.24 (1.08,1.44)	.003	1.39 (1.19,1.64)	<.0001	1.42 (1.13,1.79)	.003
NHHR category						
Q1	Ref	Ref	Ref	Ref	Ref	Ref
Q2	2.10 (1.35, 3.30)	.001	2.22 (1.38, 3.60)	.001	1.99 (1.05, 3.80)	.04
Q3	1.39 (0.90, 2.14)	.14	1.55 (0.97, 2.49)	.07	2.01 (1.05, 3.90)	.04
Q4	1.94 (1.25, 3.02)	.003	2.55 (1.55, 4.23)	<.001	2.44 (1.23, 4.92)	.01
*P* for trend	.022		.002		.015	

Model 1: no adjustments made; Model 2: adjusted for age, smoke, hypertension, BMI; Model 3: adjusted for age, smoke, diabetes, BMI, WBC, UA, FPG.

CI = confidence interval, NHHR = non-high-density lipoprotein cholesterol to high-density lipoprotein cholesterol ratio, OR = odds ratio, Ref = reference.

### 3.4. Linearity of the association

**Figure 1.**
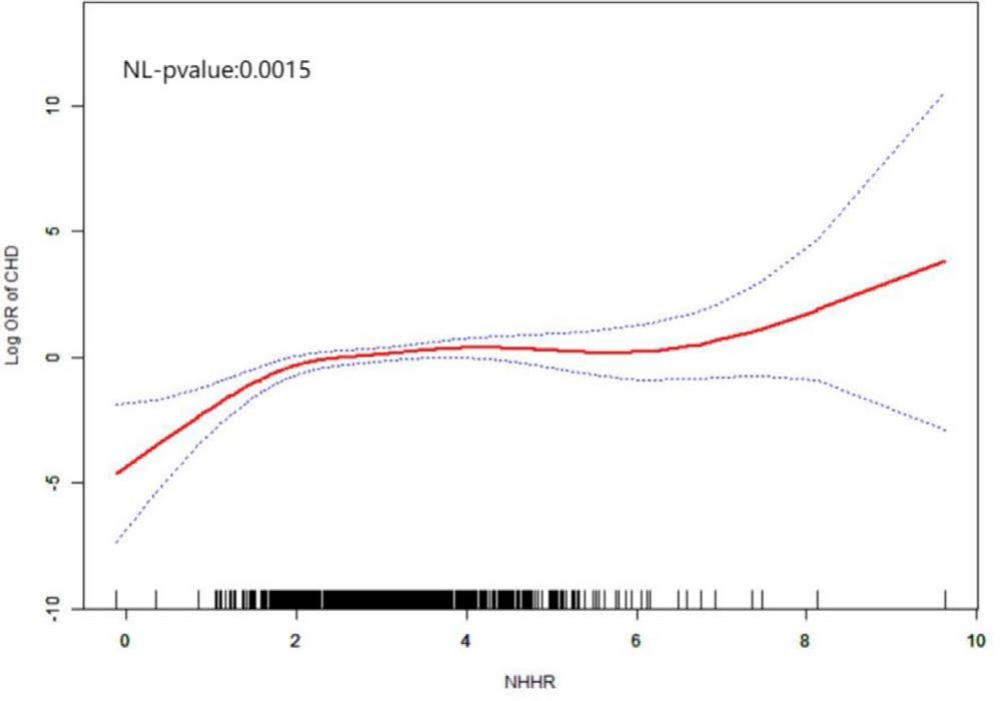
The smoothed curve fitting shows a significant nonlinear relationship between NHHR and CHD risk, indicating that as NHHR increases, the risk of CHD also increases non-linearly across the entire range of NHHR. CHD = coronary heart disease, NHHR = non-HDL cholesterol to HDL cholesterol ratio.

Table 3 shows a significant trend of increased CHD risk from Q1 to Q4. We performed a smooth curve fit to confirm whether there was a linear relationship between NHHR and CHD. The results (Fig. 1) showed that across the entire range of NHHR, the risk of CHD increased non-linearly with increasing NHHR, which was statistically validated as a non-linear relationship.

### 3.5. Establishment of nomogram model

**Figure 2.**
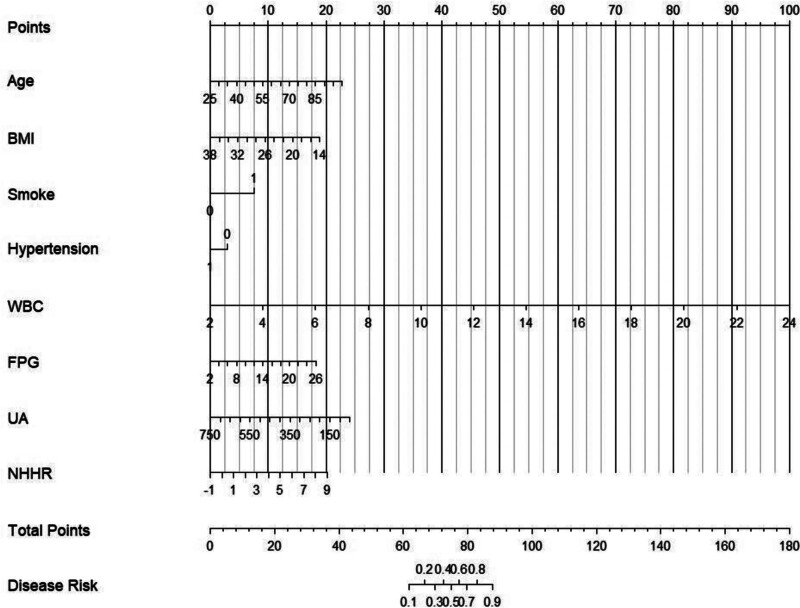
Nomogram model. BMI = body mass index, FPG = fasting plasma glucose, NHHR = non-HDL cholesterol to HDL cholesterol ratio, UA = uric acid, WBC = white blood cell.

We designed and created a Nomogram model to predict the occurrence of CHD based on independent risk factors, as shown in Figure 2. In the diagram, each predicted variable corresponds to a specific score on the horizontal axis of the line chart. By adding up the scores associated with all the predicted variables, the total score can be obtained. At the bottom of the line chart corresponding to the total score, we can see the predicted risk value of developing CHD, which increases as the total score goes higher.

### 3.6. Nomogram model validation

**Figure 3.**
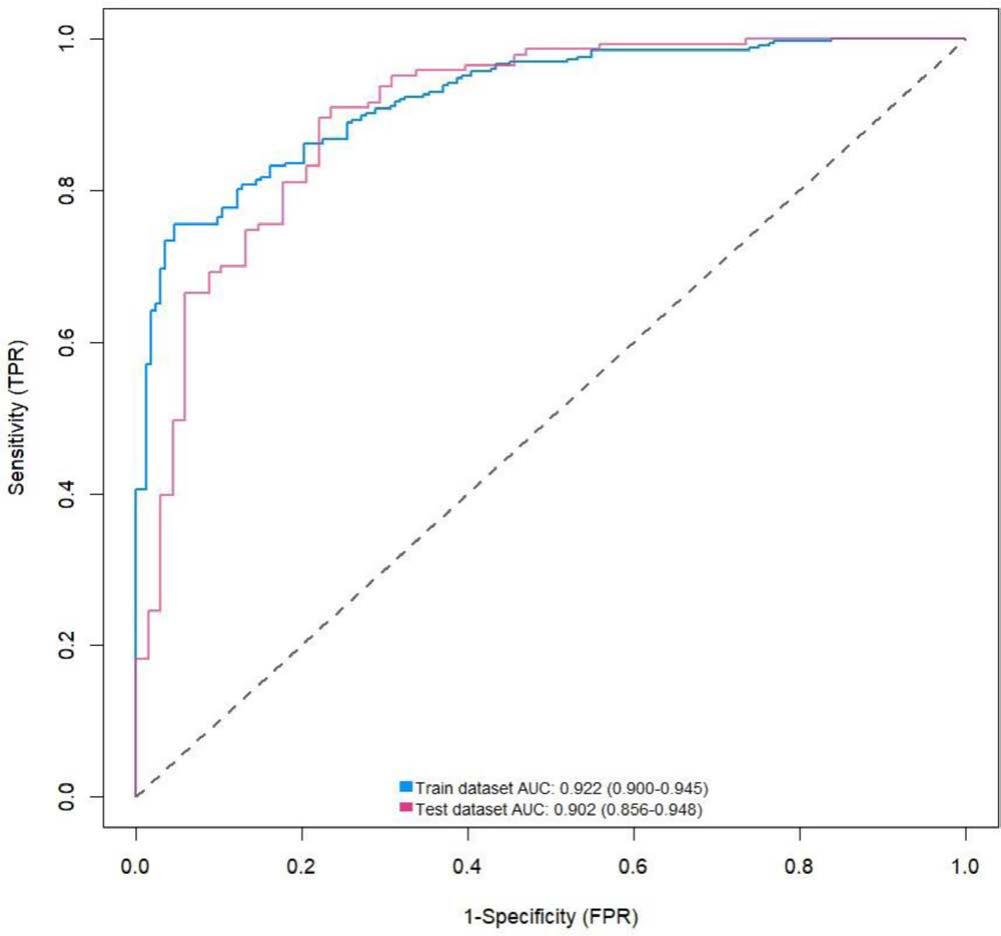
ROC curve of training set and validation set. ROC = receiver operating characteristic curve.

**Figure 4.**
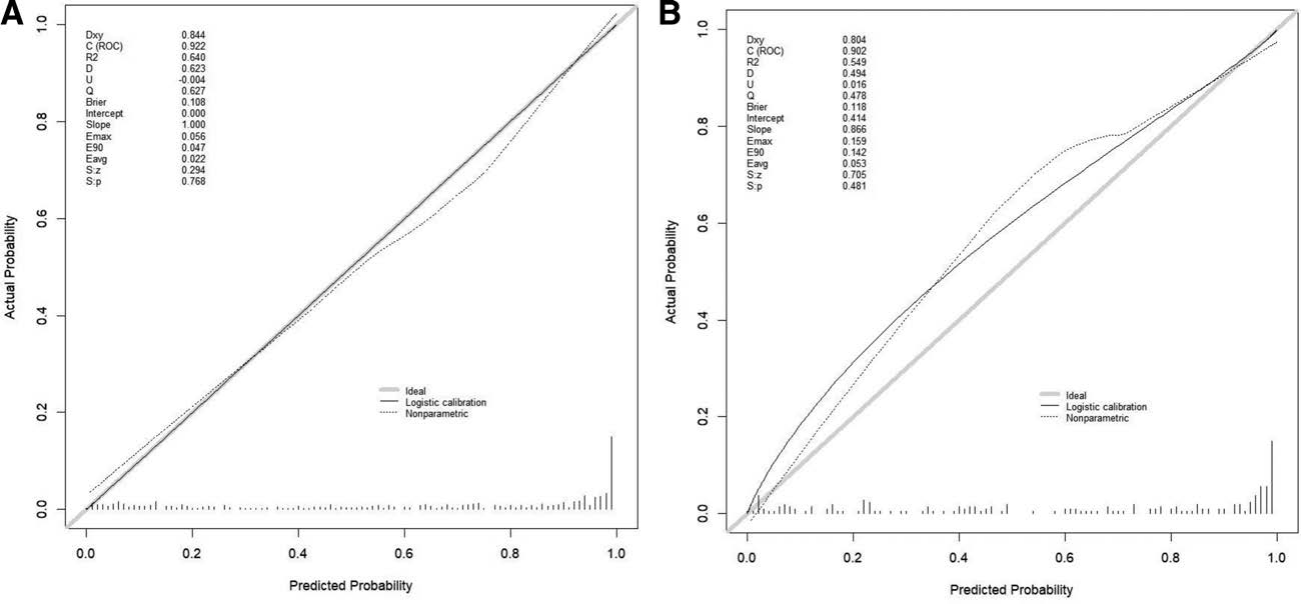
Calibration curve: (A) training set, (B) validation set.

**Figure 5.**
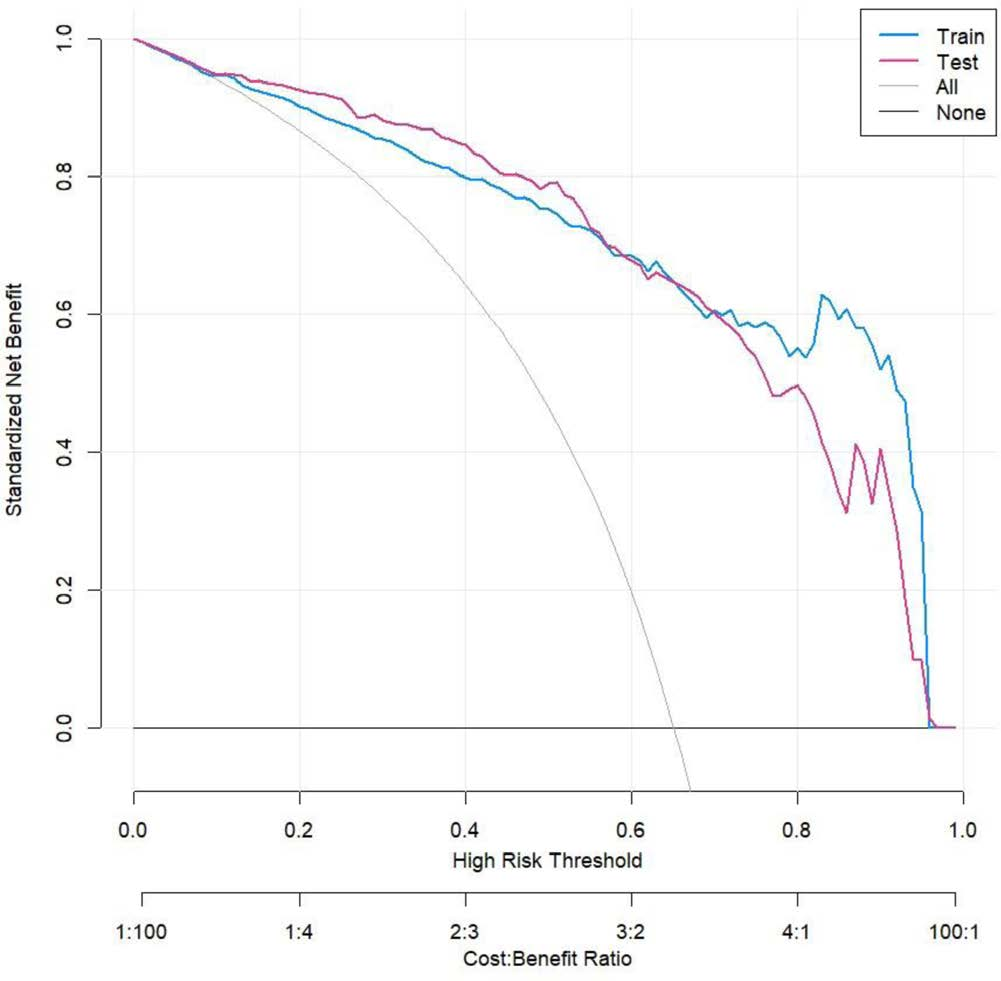
Decision curve analysis.

By analyzing the ROC curve of the training set, we found that the AUC of the model was 0.922 (95% CI: 0.900–0.945), while the AUC of the validation set was 0.902 (95% CI: 0.856–0.948), indicating that the model had good discriminatory power. The ROC curve is shown in Figure 3. Through calibration curve analysis, we found that the Nomogram model could predict the incidence of CHD in both the training and validation sets well, as evidenced by the high degree of fit between the calibration curve and the ideal curve, as shown in Figure 4. In addition, we also conducted decision curve analysis (DCA) analysis, and the results showed that this Nomogram model could bring significant clinical net benefits to patients at threshold probabilities of 0.1 to 0.95, as detailed in Figure 5.

## 4. Discussion

The results of this study demonstrate a positive correlation between NHHR levels and the risk of CHD, and NHHR is an independent predictor of coronary artery disease. Moreover, the clinical prediction model based on NHHR has high accuracy and practical value. Due to the advantages of easy accessibility and low cost, we expect NHHR to become an important biological indicator for assisting in the assessment of the risk of CHD.

In the development of coronary atherosclerosis and CHD, inflammatory reactions and abnormal lipid metabolism play crucial roles. Lipid deposition on the vascular wall, smooth muscle cell proliferation, leukocyte aggregation, and expression of proinflammatory cytokines are key steps in the process of atherosclerosis.^[12]^ Non-HDL-C mainly includes LDL, very low-density lipoprotein cholesterol, intermediate-density lipoprotein cholesterol, lipoprotein(a), and chylomicrons, among others,^[13]^ and its elevation is an important factor in the development of atherosclerosis.^[14]^ Furthermore, studies have shown that patients with higher levels of non-HDL-C have a greater risk of future CHD.^[15]^ A large prospective study has indicated that compared with patients with non-HDL-C levels < 2.6 mmol/L, those with non-HDL-C levels ≥ 5.7mmol/L have significantly higher rates of cardiovascular disease, with the incidence increasing from 7.7% to 33.7% for women and from 12.8% to 43.6% for men.^[16]^ The results of this study show that the NHHR levels in the coronary artery disease group were significantly higher than those in the control group, which also suggests a possible correlation between NHHR and the occurrence of coronary artery disease.

Non-HDL-C represents the cholesterol carried by apoB-containing particles and indicates the lipid content that is rich in triglycerides (TG). This includes LDL-C, intermediate-density lipoprotein cholesterol, very low-density lipoprotein cholesterol and its remnants, chylomicrons, and lipoprotein(a). TG-rich lipids contribute to the development of atherosclerosis through various mechanisms, such as penetrating the sub-endothelial layer of arteries and being taken up by macrophages, which leads to foam cell formation and the progression of atherosclerosis.^[1]^ Guidelines recommend placing particular emphasis on Non-HDL-C in individuals with diabetes, obesity, or metabolic syndrome, as these groups often exhibit elevated Non-HDL-C and TG levels, alongside reduced HDL-C levels, while LDL-C levels may not be significantly high.^[3,17–19]^Additionally, calculating Non-HDL-C is straightforward, requiring only the subtraction of HDL-C from TC, and it is not influenced by fasting status. This simplicity enhances convenience for patients and ensures high reliability, as it remains unaffected by variations in TG levels.

The Non-HDL-C/HDL-C ratio offers a superior representation of the balance between pro-atherogenic and anti-atherogenic lipids, reflecting a more comprehensive view of lipid metabolism imbalances. Research indicates that TC and LDL-C levels tend to decline with age in patients experiencing acute coronary syndrome.^[9]^ This suggests that applying the same reference values for LDL-C or Non-HDL-C across different age groups may not be appropriate. Consequently, utilizing lipid ratios, particularly the Non-HDL-C/HDL-C ratio, could yield a more objective evaluation of the onset, severity, and prognosis of CHD. Studies have demonstrated that the Non-HDL-C/HDL-C ratio is more effective than LDL-C and Non-HDL-C in predicting atherosclerotic disease.^[20]^ Additionally, it provides a more accurate prediction of insulin resistance and metabolic syndrome compared to the ApoB/ApoA1 ratio, likely due to disruptions in HDL-C levels.^[7]^

HDL-C has anti-atherogenic effects, as it can remove cholesterol from macrophages in the coronary artery wall and transport it to liver cells, while also having anti-inflammatory and antioxidant effects.^[21]^ NHHR is a comprehensive lipid index that integrates pro-atherogenic and anti-atherogenic lipid components and reflects lipid metabolism more comprehensively than individual lipid components. A cross-sectional study of 1626 patients with acute myocardial infarction confirmed that high NHHR is closely related to the risk of early onset of acute myocardial infarction, indicating that dyslipidemia promotes the formation and development of atherosclerosis.^[22]^ In a cohort study conducted by Mao et al in 2023, which included 426 patients with non-ST segment elevation acute myocardial infarction, NHHR was not only correlated with the severity of coronary artery lesions in these patients but also an independent predictor of major cardiovascular events.^[23]^ Liu et al found that NHHR has predictive value for the progression of non-culprit coronary artery lesions in patients with acute coronary syndrome treated with percutaneous coronary intervention (OR = 1.45).^[24]^ Studies have found that NHHR is associated with an increased risk of CHD incidence (OR = 1.291), high Gensini scores (OR = 1.408), and multi-vessel disease (OR = 1.487), indicating that NHHR is significantly correlated with the severity and progression of coronary artery disease,^[25]^ similar to the results of this study. Our findings show that the NHHR levels were significantly higher in the coronary artery disease group than in the control group (*P* < .05), and multivariate logistic regression analysis indicated that NHHR was an independent predictor of CHD risk. For each unit increase in NHHR, the risk increased by 42% (OR: 1.42, 95% CI: 1.13–1.79, *P* = .003). In the quartile analysis, compared to the lowest quartile in the entire model, NHHR in the highest quartile increased the risk by 144%. The smooth curve fitting showed a non-linear increase in CHD risk with increasing NHHR across the entire range of NHHR. Based on independent risk factors, a prediction model for CHD occurrence was further plotted. ROC curve analysis showed that the model had good discriminative ability, while calibration curve and DCA analysis indicated the model had good clinical significance for predicting the risk of CHD occurrence.

This study has the following limitations: retrospective design with lower reliability than prospective study design; small sample size; the control group consisted of patients who were excluded from CHD through coronary angiography, and patients who are clinically recommended for coronary angiography to confirm diagnosis usually have significant CHD risk factors, so this part of patients is not a completely blank control, which may cause certain bias in the study results.

Our study results indicate that NHHR measurement can serve as an effective tool to predict CHD, as it can identify the risk of CHD through a simple and cost-effective method. These findings provide increasing evidence for the potential use of biomarkers in predicting CHD and can also provide important information for clinical decision-making in the management of CHD patients.

## Author contributions

**Conceptualization:** Wenlong Ding, Tao Li.

**Formal analysis:** Caoyang Fang.

**Methodology:** Xin-Xin Zhang.

**Writing – original draft:** Wenlong Ding, Tao Li.

**Writing – review & editing:** Wenlong Ding, Tao Li, Enyang Wang.

## Supplementary Material


