# Supplementary material for: Association of non-high-density lipoprotein cholesterol to high-density lipoprotein cholesterol ratio with coronary heart disease: Establishment and validation of a clinical nomogram model
Source: Medicine (Baltimore). 2025 Mar 14;104(11):e41896. doi: 10.1097/MD.0000000000041896 (PMC11922409; doi:10.1097/MD.0000000000041896)
Supplement: Supplementary file 1 [file medi-104-e41896-s001.docx]

**Supplementary Table 1.Baseline data comparison between training set and validation set**

| **Variables** | **Total (n = 707)** | **validation set**  **(n = 213)** | **training set (n = 494)** | ***t/Z/χ²*** | ***P*** |
| --- | --- | --- | --- | --- | --- |
|  |  |  |  |  |  |
| **RBC, Mean ± SD** | 4.46 ± 0.57 | 4.48 ± 0.59 | 4.46 ± 0.56 | 0.45 | 0.651 |
| **Age, M (Q₁, Q₃)** | 62.00 (52.00, 71.00) | 61.00 (52.00, 71.00) | 63.00 (52.00, 70.00) | -0.42 | 0.677 |
| **BMI, M (Q₁, Q₃)** | 24.70 (22.47, 27.00) | 24.90 (22.80, 27.30) | 24.70 (22.30, 26.80) | -1.45 | 0.148 |
| **WBC, M (Q₁, Q₃)** | 8.54 (6.38, 11.37) | 8.84 (6.90, 11.48) | 8.34 (6.24, 11.23) | -2.00 | 0.045 |
| **Lymphocytes, M (Q₁, Q₃)** | 1.55 (1.10, 2.09) | 1.51 (1.05, 2.24) | 1.57 (1.13, 2.01) | -0.16 | 0.872 |
| **Platelets, M (Q₁, Q₃)** | 197.00 (156.00, 239.50) | 195.00 (156.00, 249.00) | 197.00 (156.12, 235.75) | -0.66 | 0.510 |
| **FPG, M (Q₁, Q₃)** | 5.79 (5.08, 7.37) | 5.97 (5.18, 7.48) | 5.69 (5.04, 7.25) | -2.00 | 0.046 |
| **BUN, M (Q₁, Q₃)** | 5.45 (4.43, 6.75) | 5.49 (4.61, 6.60) | 5.43 (4.40, 6.79) | -0.53 | 0.593 |
| **Creaintine, M (Q₁, Q₃)** | 69.00 (58.75, 83.90) | 70.00 (57.10, 86.70) | 69.00 (59.00, 82.18) | -0.38 | 0.703 |
| **UA, M (Q₁, Q₃)** | 357.60 (294.90, 427.55) | 367.90 (293.60, 434.10) | 353.60 (296.00, 425.75) | -0.73 | 0.468 |
| **TG, M (Q₁, Q₃)** | 1.43 (1.01, 2.07) | 1.40 (1.00, 1.98) | 1.44 (1.02, 2.09) | -0.82 | 0.409 |
| **LDL, M (Q₁, Q₃)** | 2.77 (2.24, 3.39) | 2.71 (2.24, 3.42) | 2.80 (2.24, 3.38) | -0.53 | 0.595 |
| **NHHR, M (Q₁, Q₃)** | 3.07 (2.34, 3.73) | 3.00 (2.29, 3.73) | 3.10 (2.35, 3.73) | -0.58 | 0.565 |
| **Sex, n(%)** |  |  |  | 0.15 | 0.701 |
| Female | 176 (24.89) | 51 (23.94) | 125 (25.30) |  |  |
| Male | 531 (75.11) | 162 (76.06) | 369 (74.70) |  |  |
| **Smoke, n(%)** |  |  |  | 2.94 | 0.086 |
| No | 360 (50.92) | 98 (46.01) | 262 (53.04) |  |  |
| Yes | 347 (49.08) | 115 (53.99) | 232 (46.96) |  |  |
| **Alcohol, n(%)** |  |  |  | 1.46 | 0.227 |
| No | 545 (77.09) | 158 (74.18) | 387 (78.34) |  |  |
| Yes | 162 (22.91) | 55 (25.82) | 107 (21.66) |  |  |
| **Diabetes, n(%)** |  |  |  | 0.03 | 0.858 |
| No | 558 (78.93) | 169 (79.34) | 389 (78.74) |  |  |
| Yes | 149 (21.07) | 44 (20.66) | 105 (21.26) |  |  |
| **Hypertension, n(%)** |  |  |  | 1.89 | 0.170 |
| No | 288 (40.74) | 95 (44.60) | 193 (39.07) |  |  |
| Yes | 419 (59.26) | 118 (55.40) | 301 (60.93) |  |  |

**RBC:Red blood cell;BMI:Body mass index;WBC:White blood cell;FPG:Fasting plasma glucose;BUN:Blood urea nitrogen;UA:Uric acid;TG:Triglyceride;LDL:Low density lipoprotein;NHHR:Non-HDL cholesterol to HDL cholesterol ratio**
